# Negative-pressure wound therapy for management of chronic neuropathic noninfected diabetic foot ulcerations – short-term efficacy and long-term outcomes

**DOI:** 10.1007/s12020-018-1707-0

**Published:** 2018-08-11

**Authors:** S Borys, J Hohendorff, T Koblik, P Witek, AH Ludwig-Slomczynska, C Frankfurter, B Kiec-Wilk, MT Malecki

**Affiliations:** 1 Department of Metabolic Diseases, University Hospital in Krakow, Krakow, Poland; 2 Department of Metabolic Diseases, Jagiellonian University Medical College, Krakow, Poland; 3 Center for Medical Genomics OMICRON, Jagiellonian University Medical College, Krakow, Poland; 4 Faculty of Medicine, University of Toronto, Toronto, Canada

**Keywords:** Negative-pressure wound therapy, Diabetic foot syndrome, Type 2 diabetes

## Abstract

**Purpose:**

Negative pressure wound therapy (NPWT) is an adjunct method used in the treatment of diabetic foot ulceration (DFU). Real world data on its effectiveness and safety is scarce. In this prospective observational study, we assessed the short-term efficacy, safety, and long-term outcomes of NPWT in patients with type 2 diabetes (T2DM) and neuropathic, noninfected DFUs.

**Methods:**

Based on wound characteristics, mainly area (>1 vs. ≤1 cm^2^), 75 patients with DFUs treated in an outpatient clinic were assigned to NPWT (*n* = 53) or standard therapy (*n* = 22). Wound area reduction was evaluated after 8 ± 1 days. Long-term outcomes assessed included complete ulceration closure and recurrence rate.

**Results:**

Patients assigned to NPWT were characterized by greater wound area (15.7 vs. 2.9 cm^2^). Reduction in wound area was found in both the NPWT (−1.1 cm^2^, −10.2%, *p* = 0.0001) and comparator group (−0.3 cm^2^, −18.0%, *p* = 0.0038). No serious adverse events related to NPWT were noted. Within 1 year, 55.1% (27/49) of DFUs were closed in the NPWT group and 73.7% (14/19) in the comparator group (*p* = 0.15). In the logistic regression, wound duration and smaller initial area, but not treatment mode, were associated with closure. One-year follow-up after DFU resolution revealed an ~30.0% recurrence rate in both groups (*p* = 0.88).

**Conclusions:**

NPWT is a safe treatment for neuropathic, nonischemic, and noninfected DFU in patients with T2DM, although this observational study did not prove its effectiveness over standard therapy. Additionally, we report a high rate of both closure and recurrence of ulcers, the latter irrespective of initial ulcer area.

## Introduction

Life expectancy in people with diabetes mellitus, particularly the type 2 form of the disease, is shorter compared to the general population [1]. This is mainly attributable to its chronic complications, such as coronary artery disease, stroke, and renal failure. Diabetic foot syndrome (DFS), frequently occurring together with ulceration, is another prominent complication. The pathomechanism of DFS is complex and involves diabetic neuropathy, ischemia, and impaired function of the immune system [2]. DFS is associated with a high rate of hospitalizations and a 20-fold increase in the risk of lower limb amputations [3, 4]. Foot ulcers precede more than 80% of nontraumatic lower extremity amputations in patients with diabetes [5, 6]. DFS is also associated with increased mortality [7, 8].

In spite of new therapies for diabetes mellitus that have become available in the recent decades, DFS still affects thousands of patients worldwide and constitutes a large medical, organizational, and economic problem. There are a number of approaches in the treatment of DFS with ulceration that are used either subsequently or simultaneously, depending on the type of the wound, accessibility, and local guidelines [9]. This list includes surgical debridement of the injury bed, off-loading of the affected foot, systemic administration of wide-spectrum antibiotics when infection is present, optimization of glycemic control, and endovascular treatment (angioplasty and stenting) or surgery for peripheral artery disease if applicable. Nevertheless, in some patients, these conventional procedures are not effective, resulting in prolonged healing of foot ulcerations. Among adjuvant methods that appear to accelerate wound healing, negative-pressure wound therapy (NPWT) seems to be particularly effective in diabetic foot ulcerations. NPWT involves the use of a device that is connected to the wound bed through a special set and generates a negative pressure [10]. Proposed mechanisms of its action at the tissue and cellular level include reduction of the edema, local blood flow improvement, granulation and angiogenesis induction, epithelialization of the wound borders, and facilitation of cell migration and proliferation [10]. Macrostrain mechanisms of NPWT involve both union of wound edges and removal of exudates with infectious materials from the wound bed. NPWT has been shown to be safe and effective in wound healing, especially in postoperative lesions. Its efficacy in diabetic foot ulcers was confirmed by several inpatient randomized controlled trials [11, 12]. However, there is still a need for real world observational data from outpatient clinics concerning its use in specific ulcer subtypes.

The aims of this prospective observational study were: (1) to assess the short-term efficacy and safety data of NPWT use in patients with type 2 diabetes mellitus (T2DM) and concomitant neuropathic, nonischemic, noninfected foot wounds; (2) to collect information about long-term outcomes in these patients and compare them with patients not exposed to NPWT and patients with different wound characteristics, notably smaller ulcer areas.

## Subjects and methods

The patients included in this prospective clinical observation were participants of the research project on the molecular mechanisms of NPWT [13]. The total study group consisted of 75 consecutive patients with T2DM and concomitant foot ulceration(s) treated between 2014 and 2018 in the Department of Metabolic Diseases of the University Hospital in Krakow, a tertiary academic outpatient center for patients with DFS in southern Poland. Patients were qualified to the study if they had peripheral diabetic polyneuropathy (diagnosed with at least two standard clinical tests) complicated by superficial neuropathic, noninfected, and nonischemic foot ulcerations. Exclusion criteria included active osteomyelitis and active Charcot neuroarthropathy that were verified by clinical examination (depth of wound, probe-to-bone test) and radiographs of the affected foot, as well as clinically significant ischemia defined as the lack of pulses of both main foot arteries and/or an ankle-brachial index <0.9. Subjects were assigned to the standard therapy alone or combined with NPWT for 8 ± 1 days, a duration routinely used in our clinical practice. Allocation of patients with T2DM to study arms was not random but based on wound characteristics and pre-specified criteria. Patients were assigned to the NPWT group if they had neuropathic, noninfected ulcerations of an area >1 cm^2^ on one foot, or to the comparator group if they had ulcerations of an area ≤1 cm^2^ or bilateral DFS. Conversely, patients were allocated to the comparator group, if they had ulcerations of an area >1 cm^2^, if technical difficulties existed (e.g. unfavorable localization for NPWT application), or if the patient did not consent to NPWT. At the initial visit, patients were assigned to treatment including, or not including, the use of NPWT. The NPWT dressing was changed 3–5 days after the initial visit, and after another 3–5 days, the treatment was terminated. Patients assigned to the comparator group had control visits at the same time intervals. At each timepoint, a standard clinical examination and wound debridement was performed. We used a commercial NPWT system (Renasys EZ Plus/Smith & Nephew, London, United Kingdom) with a portable device and adjusted fluid canister. Continuous negative pressure of −120 mmHg was applied during therapy. Renasys F foams were cut and fitted to the wound bed, and Renasys soft ports were used as above when dressings were changed. The study protocol was approved by the Jagiellonian University Bioethical Committee and was in accordance with the Declaration of Helsinki. Informed consent was obtained from all individual participants included in the study.

After this initial time period, all subjects remained under routine medical care and received standard therapy in our center. We performed a follow-up to assess long-term results of ulcer healing and potential ulcer recurrence in both study groups with different baseline ulcer characteristics. After the initial phase, all patients remained under routine outpatient care. During the whole period of treatment and after wound closure, telephone contact with the clinic center was also possible for patients. A standard off-loading method through the use of appropriate footwear, two crutches and/or a wheelchair was advised during therapy. Moreover, basic rehabilitation techniques with a minimal wound damage risk profile were advised. For homecare, patients were also advised to change a standard wound dressings two times a day after typical basic hygiene procedures using sterile gauze and saline and/or colorless antiseptic fluids. During both the initial and follow-up phases, ulcer area was measured at every visit using the MOWA Mobile Wound Analyzer application (Healthpath, Italy).

For the initial phase, we defined the main outcome as a change in wound area within each study group expressed as an absolute value, as well as a percentage. The list of outcomes for the follow-up phase included closure and recurrence rate in each group, as well as the comparison between groups. A full epithelialization of the wound bed without the presence of discharge or crust was required to consider the ulcer closed. We also followed deaths and large amputations.

Statistical analysis was performed using Statistica Software v. 13.0 (TIBICO Software, Palo Alto, CA, USA). A *p* value of <0.05 was considered significant. Parametric *t* tests or nonparametric *U* tests were performed to describe baseline clinical characteristic of the study groups. To assess changes in wound areas after treatment within study groups, Wilcoxon tests were performed. Clinical factors affecting the closure within 12 months in the study groups were assessed with logistic regression. The list of independent variables consisted of age, sex, BMI, initial mode of treatment (NPWT vs. standard), wound duration, wound area, diabetes duration, HbA1c value, kidney function (GFR), and smoking status.

## Results

Overall, we included 75 patients with T2DM in this observation. There were 53 patients assigned to the NPWT group and 22 to the comparator group. Most of patients were assigned to the comparator group based on the wound area (ulcerations ≤1 cm^2^), rather than other criteria. No differences between the study groups in terms of baseline characteristics were found, as summarized in Table 1. The NPWT group was characterized by greater wound areas than in the comparator group (Table 2), which was in line with how patients were allocated. Mean wound area was 15.7 ± 14.6 cm^2^ in the NPWT group and 2.9 ± 4.4 cm^2^ in the comparator group. However, after removal of 4 outliers with a large ulcer area, the mean wound area in comparator group was 1.6 ± 1.2 cm^2^.

**Table 1:** Baseline clinical characteristics and biochemical measurements of the study groups

	NPWT group	Comparator group	*p* value
Number of cases; *n*	53	22	NA
Sex; *n* M/F, % M	45/8, 84.9%	17/5, 77.3%	0.42
Age; years^a^	65.4 ± 8.6	64.2 ± 6.8	0.54
Duration of diabetes; years^a^	14.3 ± 8.0	17.1 ± 7.4	0.17
BMI; kg/m^2 a^	29.8 ± 5.1	30.5 ± 4.9	0.60
Insulin therapy; *n* Y/N, %Y	50/3, 94.3%	20/1, 95.2%	0,87
Daily dose of insulin; U^a^	55.3 ± 27.5	55.0 ± 25.9	0.97
HbA1c; %^a^	7.0 ± 1.3	7.4 ± 1.5	0.30
HbA1c, mmol/mol^a^	53.5 ± 13.9	57.4 ± 16.8	0.30
eGFR CKD EPI; ml/min/1,73 m^2 a^	79.6 ± 21.7	74.5 ± 21.1	0.35
eGFR < 60 ml/min/1,73 m^2^, % (*n*)	20.8% (11)	31.8% (7)	0.31
Total cholesterol; mmol/l^a^	3.3 ± 0.8	3.4 ± 0.9	0.75
HDL; mmol/l^a^	0.9 ± 0.4	0.8 ± 0.1	0.90
LDL; mmol/l^a^	2.0 ± 0.8	2.1 ± 0.7	0.45
Triglycerides; mmol/l^a^	1.3 ± 0.6	1.5 ± 0.7	0.60
Smoking; *n* current/former/never	4/27/18	3/9/8	0.61

^a^Data presented as mean ± SD

**Table 2:** Wound-related study characteristics and outcomes

	NPWT group	Comparator group	*p* value
Wound duration, weeks^a^	21.1 ± 24.7	14.4 ± 14.3	0.10
Wound area; cm^2 a^	15.7 ± 14.6	2.9 ± 4.4	0.0000
Wound area reduction after 8 ± 1 days; cm^2 a^	1.1 ± 1.7	0.3 ± 0.7	0.20^b^
Wound area reduction after 8 ± 1 days; %^a^	10.2 ± 14.41	18.0 ± 15.7	0.04^b^
Complete ulcer resolution within 1 year; % (n)	55.1 (27/49)	73.7 (14/19)	0.15
Ulcer recurrences within 1 year after complete ulcer resolution; % (*n*)	29.2 (7/24)	31.3 (5/16)	0.88
Median follow-up, months, mean SD, median, IQ range	21.1 ± 9.1 21.1 (IQR: 15.9–28.3)	23.8 ± 10.4 29.8 (IQR: 15.2–31.7)	0.26
Large amputations; *n*	0	1	0.11
Deaths; *n*	3	2	0.61

^a^Data presented as mean ± SD

^b^The *p*-value for the reduction in wound area after 8 ± 1 days in the NPWT was *p* = 0.0001, while in the comparator group it was 0.0038

Reduction in wound area after 8 ± 1 days was found in both the NPWT and comparator group (−1.1 ± 1.7 cm^2^, −10.2% ± 14.4%, *p* = 0.0001 and −0.3 ± 0.6, −18.0% ± 15.7%, *p* = 0.0038, respectively). During the initial phase, we did not note either local or systemic serious complications with NPWT, including pain, cellulitis, infections, necrosis, hemorrhage, and thrombosis. There were 2 patients from the NPWT group and none from comparator group that were lost to follow-up at the end of the initial phase of the study.

**Fig. 1:**
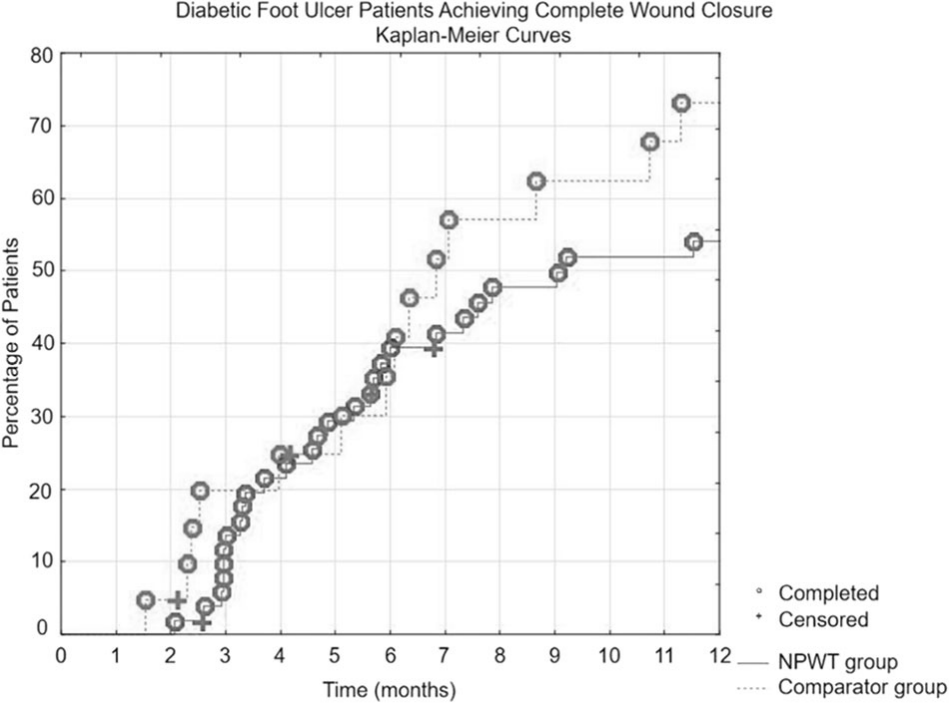
Kaplan–Meier survival curves showing percentage of patients with diabetic foot syndrome achieving complete wound closure

After the initial phase, all patients were observed on average every 5.2 ± 2.6 weeks until the whole ulceration was closed. Within 1 year, 55.1% (27/49) of foot ulcerations were healed in the NPWT group and 73.7% (14/19) in the comparator group (*p* = 0.1525). During the median follow-up (time to complete ulceration closure or time to loss of follow-up) of 7.6 months (IQR: 4.1–17.3 months) in NPWT group and 6.3 months (IQR: 4.0–11.3 months), a complete ulcer closure was achieved in 78.4% (40/51) and 77.3% (17/22) patients, respectively (*p* = 0.9126). Based on the logistic regression, the factors associated with complete wound closure within one year among the pooled group were shorter wound duration (OR: 0.885, 95% CI: 0.799–0.982) and smaller wound area (OR: 0.905, 95% CI: 0.830–0.986), but not other factors, including mode of treatment. Kaplan-Meier survival curves are shown in Fig. 1. One-year follow-up after ulceration resolution revealed 29.1% (7/24) and 31.3% (5/16) of recurrences in foot ulceration in the NPWT and comparator group, respectively (*p* = 0.8880). During the median entire follow-up of 21.8 months (IQR: 15.6–29.9), a large amputation occurred in one patient and 5 patients died (3 of them after complete wound healing; 3 deaths occurred in patients from the NPWT group and 2 from the comparator group). Moreover, there were 5 patients with unhealed wounds lost to follow-up (4 patients from the NPWT group after 3, 7, 15, and 16 months and 1 patient from comparator group after 4 months). Additionally, 8 patients were lost to follow-up after complete ulcer healing (mean 4.3 ± 4.8 months) Treatment outcomes are summarized in Table 2.

## Discussion

We present the results of a prospective clinical outpatient observation of patients with T2DM and concomitant foot ulcers. We report the effective initial, rapid response to NPWT in patients with neuropathic, nonischaemic, and noninfected foot wounds. Additionally, long-term follow-up data with a rate of both healing and recurrence are provided for ulcers of different areas.

Patients with neuropathic, nonischemic, and noninfected plantar ulcerations constitute approximately one-quarter of patients in diabetic foot clinics [14]. While closure is reached within 1–2 months in a number of affected individuals, the remaining patients experience prolonged healing and constitute a real challenge in clinical practice. The International Working Group on the Diabetic Foot (IWGDF) suggests using a nonremovable total contact cast (TTC) that has shown to be of benefit in wound healing for diabetic ulcerations with such characteristics [15–17]. However, due to its time-consuming nature and infrequent possibility for reimbursement, amongst other causes, TCC is applied only in 6% of patients who are potentially good candidates for such a treatment [18]. NPWT is another option recommended for the treatment of diabetic foot ulcers by some international expert groups [19]. Its efficacy was confirmed in 2 long-term randomized trials, both of them lasting 112 days, where it showed superiority over standard approaches or advanced moist wound therapy in the treatment of diabetic foot ulcers, reaching closure rates of 43 and 56% in the two trials, respectively [11, 12]. Evidence pertaining its efficacy in the real world has nonetheless been scarce.

In our study, a reduction of approximately 10% of the wound area was reached after 8 days of exposure to NPWT. This was similar to a 12% decrease reached in a similar study with a duration twice the length of ours [20]. It should be noted that due to the observational nature of this study, we were not able to fully objectively compare the effectiveness of NPWT over standard therapy. Unlike the Israeli study, no serious adverse events were recorded in this trial.

In our study, 55% of ulcers in the NPWT group and over 70% in comparator group were completely healed within one year. Data analyses from 14 centers in Europe performed within the EURODIALE study showed a similar ulcer resolution rate [21]. The list of independent clinical predictors of complete ulcer healing includes time since the beginning of the ulceration and the magnitude of its area. Of note, the HbA1c level did not influence this outcome, in line with a recently published observation [22]. Furthermore, it is well-known that end-stage renal failure and dialysis are associated with poor wound healing and limb amputation. There are also studies showing impaired wound healing in subjects with moderately reduced eGFR [23]. In our study, patients with eGFR < 60 ml/min/1,73 m^2^ accounted for 24% of study group and eGFR was not found to be associated with healing failure. One-year follow-up revealed an ulcer recurrence rate of over 30%, a finding in line with other studies [24]. Interestingly, the rate of ulcer recurrence was almost the same in both groups, in spite of a large difference in the magnitude of their initial area. This finding should alert clinicians to the fact that a high rate of recurrence is present also in ulcers of relatively small area. It has been in fact previously proposed that clinicians use the terms “ulcer remission” rather than “ulcer healing” in clinical practice [24, 25]. In the presented study, following ulcer closure, all patients received standard advice on foot ulcer recurrence prevention (e.g. maintaining adequate glycemic control, using appropriate footwear, and inspecting the foot each day). However, there is no certainty that even targeted, structured educational programs are associated with clinical benefits [26]. A population-based study revealed over a 2-fold increase in the mortality risk among individuals with a history of foot ulcers [27]. The mortality risk among patients with lower limb amputation is comparable to those with malignant diseases [8]. Due to the poor prognosis associated with diabetic foot ulcers and lower limb amputations, a greater emphasis on primary and secondary prevention is needed within healthcare systems.

There are several shortcomings related to this study. First of all, the study is limited by its nonrandom, observational nature. Subsequently, there were substantial differences in the magnitude of ulcer areas in both study groups. Additionally, use of more reliable methods to exclude a substantial ischemic component of an ulcer, such as transcutaneous oxygen pressure measurement or toe systolic pressure measurement, was not available for this study. Many known and likely unknown risk factors were not included in the regression analysis. Moreover, 20% of patients were lost to follow-up, as some of them likely decided to return to their local outpatient clinics, particularly after they experienced closure of their ulcerations. However, we cannot exclude the possibility that some of them had ulcer recurrences, underwent amputations, or died. Despite these limitations, our study exhibited important real-world data on treatment of diabetic plantar ulcerations.

## Conclusions

In summary, NPWT is safe for the treatment of neuropathic, nonischemic, and noninfected plantar ulcerations in patients with T2DM although this observational study did not prove its better effectiveness over standard therapy. Additionally, we report a high rate of both closure and recurrence of ulcers, the latter irrespective of initial ulcer area.
